# Newborn and Infant Hearing Screening Facing Globally Growing Numbers of People Suffering from Disabling Hearing Loss

**DOI:** 10.3390/ijns5010007

**Published:** 2019-01-18

**Authors:** Katrin Neumann, Shelly Chadha, George Tavartkiladze, Xingkuan Bu, Karl R. White

**Affiliations:** 1 Department of Phoniatrics and Pediatric Audiology, Clinic of Otorhinolaryngology, Head and Neck Surgery, Ruhr-University of Bochum, St. Elisabeth-Hospital, Bleichstr. 16, 44787 Bochum, Germany; 2 Blindness, Deafness Prevention, Disability and Rehabilitation Unit, Department for Management of Noncommunicable Diseases, Disability, Violence and Injury Prevention, World Health Organization, 20 Avenue Appia, 1211 Geneva 27, Switzerland; 3 Department of Physiology and Pathology of Hearing, National Research Centre for Audiology and Hearing Rehabilitation, 123 Leninsky ave, Moscow 117513, Russia; 4 WHO Collaborating Center for the Prevention of Deafness and Hearing Impairment, Nanjing Medical University, Nanjing 210029, China; 5 National Center for Hearing Assessment and Management, Utah State University, 2615 Old Main Hill, Logan, UT 84322, USA

**Keywords:** hearing loss, children, newborn hearing screening, neonatal hearing screening, infant hearing screening

## Abstract

Recent prevalence estimates indicate that in 2015 almost half a billion people—about 6.8% of the world’s population—had disabling hearing loss and that prevalence numbers will further increase. The World Health Organization (WHO) currently estimates that at least 34 million children under the age of 15 have disabling hearing loss. Based on a 2012 WHO report, approximately 7.5 million of these children were under the age of 5 years. This review article focuses on the importance of high-quality newborn and infant hearing screening (NIHS) programs as one strategy to ameliorate disabling hearing loss as a global health problem. Two WHO resolutions regarding the prevention of deafness and hearing loss have been adopted urging member states to implement screening programs for early identification of ear diseases and hearing loss in babies and young children. The effectiveness of these programs depends on factors such as governmental mandates and guidance; presence of a national committee with involvement of professionals, industries, and stakeholders; central oversight of hearing screening; clear definition of target parameters; presence of tracking systems with bi-directional data transfer from screening devices to screening centers; accessibility of pediatric audiological services and rehabilitation programs; using telemedicine where connectivity is available; and the opportunity for case discussions in professional excellence circles with boards of experts. There is a lack of such programs in middle- and low-income countries, but even in high-income countries there is potential for improvement. Facing the still growing burden of disabling hearing loss around the world, there is a need to invest in national, high-quality NIHS programs.

## 1. Introduction

Hearing loss has become the fourth leading cause of disability globally [1,2]. Disabling hearing loss impairs interpersonal communication, psychosocial wellbeing, academic and professional career opportunities, economic independence, and quality of life. According to the World Health Organization (WHO), a hearing loss is considered to be disabling for adults (15 years or older) if it is greater than 40 dB in the better hearing ear. For children (0 to 14 years), the WHO has defined a hearing loss exceeding 30 dB in the better hearing ear as disabling. However, hearing loss at lower thresholds also has a negative impact. For example, children whose hearing loss exceeds 26 dB have trouble understanding soft speech from a distance or in background noise [3].

**Figure 1:**
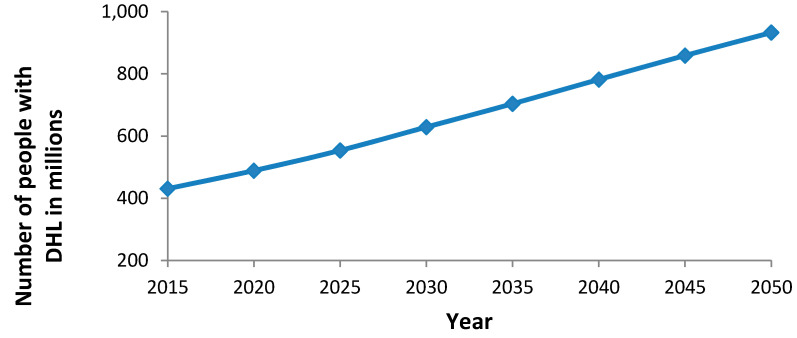
Global increase (estimated and projected) of number of people with disabling hearing loss (modified according to [1]).

The number of people in the world who suffer from a disabling hearing loss has increased from 42 million in 1985 to 360 million in 2010 [4]. Seven and a half million of these people were younger than 5 years of age. Recent estimates show a further increase to 466 million people with disabling hearing loss as of the current time [5]. These people make up over 6% of the world’s population [1]. Thirty-four million (7.3%) of the 466 million people with disabling hearing loss are children, with the highest numbers in South Asia, Asia Pacific and Sub-Saharan Africa. These absolute numbers have increased more than estimated and are expected to continue to increase during the next years [1,6,7], mainly due to the growth and the aging of the world´s population. It is predicted that without counter-measures, as many as 630 million people could suffer from disabling hearing loss by 2030 and nearly 900 million by 2050 [5,8] (Figure 1).

In a recent Lancet publication, Wilson and co-authors [7] noted that hearing loss was the 11th leading cause of years lived with disability (YLDs) in 2010 and the fourth leading cause in both 2013 and 2015. Worldwide prevalence estimates for all hearing loss ≥20 dB increased from 14.3% in 1990 to 18.1% in 2015, and estimates for disabling hearing loss increased from 5.7% in 2005 to 6.4% in 2015 [7]. All these figures indicate that the burden of hearing loss is growing and that it has become a major concern for global health.

This alarming situation has developed despite increased knowledge about the substantial health care burden caused by poorly treated or untreated hearing loss. Unfortunately, permanent hearing loss is generally underestimated by the public health administrators and politicians and is not a high priority in most countries [7,8,9]. This neglect, and the associated poor resource allocation, might be related to the fact that much of hearing loss is a non-obvious, “hidden” condition [7,10].

For children, prevention has been identified as the most effective strategy to reduce disabling hearing loss in countries with low economic prosperity [7], whereas for high-income countries universal newborn hearing screening has been proven to be a leading opportunity [7,11,12,13]. Even if the aging of the world’s population is a major contributor to the increased prevalence of hearing loss, implementation of universal newborn hearing screening in a country may sensitize the society to the problem of disabling hearing loss and increase public awareness of its negative consequences. It may also influence the willingness of older children and adults to participate in hearing screenings and to use assistive listening devices. Additionally, there is not only an increased proportion of people with hearing loss (increased prevalence), but also a higher absolute number due to an increasing world population. This affects hearing screening programs directly.

Common risk factors for hearing loss, most of which are relevant to newborns and young children, are ear infections (in particular, chronic suppurative otitis media), rubella, measles, mumps, meningitis, congenital cytomegalovirus, ototoxic agents, and exposure to occupational or recreational noise. Some of these factors have become less common during recent years in some regions of the world (e.g., rubella measles, mumps, meningitis), while others are increasing (e.g., exposure to loud recreational noise). Eighty percent of people with disabling hearing loss live in low- or middle-income countries [14], where chronic otitis media, poor ear hygiene, poor birth conditions, and lack of vaccination programs contribute substantially to the incidence of permanent childhood hearing loss. Moreover, the global production of hearing aids covers less than 3% of the needs in these countries [15]. Further barriers include insufficient services for fitting, repair, and maintenance of hearing technology, and a lack of financial resources.

The WHO has made a sustained effort for many years to prevent, identify, and treat disabling hearing loss. In 1995 the World Health Assembly passed a resolution that urged member states to prepare national plans for the prevention and control of major causes of avoidable hearing loss, and for early detection of hearing loss in babies, toddlers, and children, as well as in the elderly, within the framework of primary health care [16]. However, over the next several decades only 32 member states developed policies and plans to improve hearing health care. Additionally, a 2013 WHO report noted an overall scarcity of epidemiological data regarding hearing loss [17].

Since the 1995 resolution, WHO has made several efforts to promote a common understanding about the concepts, mechanisms, and strategies for providing appropriate hearing health care and to develop and establish instruments for planning, implementation, and monitoring hearing health care programs in individual countries [9]. Expert groups convened by WHO have also developed several tools to support decision makers in the WHO member states. These tools include brochures about the nature and reasons of childhood hearing loss and strategies for its prevention and care [18], summaries of the global costs of unaddressed hearing loss and the cost–benefits of various interventions [19], a situation analysis tool for ear and hearing care [20], and a manual on planning and monitoring national strategies for improving hearing health care [21]. Acknowledging the growing impact of disabling hearing loss, the World Health Assembly (WHA) approved a new resolution in 2017 (WHA 70.13) [22] that calls on governments and stakeholders in the field of ear and hearing care to initiate action for the prevention of deafness and hearing loss.

## 2. Prevention of Childhood Hearing Loss

**Figure 2:**
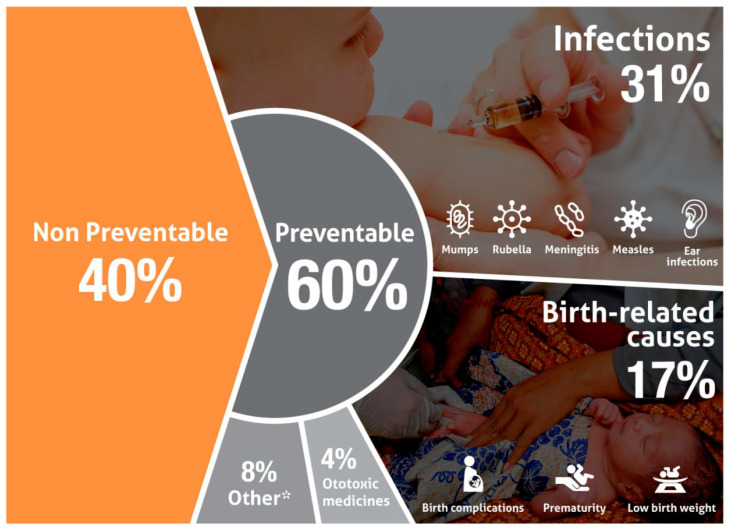
Causes of preventable hearing loss. Other causes include congenital non-genetic malformations and other maternal prenatal causes (from: [18]).

According to recent WHO estimates, more than 60% of hearing loss in children could be prevented—75% in middle- and lower-middle-income countries, 49% in high-income regions [18] (Figure 2). The difference between middle- and lower-middle-income countries in the proportion of hearing loss due to preventable causes is most probably due to a higher incidence of infections, such as congenital cytomegalovirus infections, in lower-middle-income countries, as well as fewer programs to support maternal and child health in lower-income countries compared to higher-income ones. As shown in Figure 2, about one third of cases of preventable infant hearing loss have infectious causes, such as rubella and meningitis, and an additional 4% are caused by the use of ototoxic medication. Birth complications, in particular hypoxemia, low birthweight, neonatal hyperbilirubinemia, and prematurity account for 17% of preventable hearing loss [18].

Effective strategies for preventing hearing loss include reducing the incidence of infections such as rubella, meningitis, cytomegalovirus, mumps, measles, and otitis through immunization, hygienic measures, timely medical and surgical treatment, mother and child health programs, and reductions in the use of ototoxic agents [18]. For example, the risk of a cochlear injury from ototoxic antibiotic medications with aminoglycosides in the fetal, neonatal, and infant periods is especially high, depending on application, dosage, previous aminoglycoside treatment, kidney function, pre-injury of the inner ear (e.g., by syndromes or asphyxia), presence of anemia, familial and individual sensitivity, age, environmental noise, and combination with other ototoxic or nephrotoxic medication [23]. Furthermore, there are indications that the treatment of babies with aminoglycosides in noisy neonatal care units may lead to hair cell damage and hearing loss [24]. Some mitochondrial DNA mutations also increase the risk of an aminoglycoside-induced hearing loss [25]. All these factors have to be considered before using these ototoxic antibiotics in neonatal care. As another example, the incidence of congenital cytomegalovirus infections can be reduced by counselling expectant mothers about the importance of frequent handwashing and avoiding contact with the child’s saliva, urine, and nasal secretions.

## 3. Relevance and Principles of Newborn Hearing Screening

Sequelae of untreated or late-treated infant hearing loss are deficits of language, cognitive, social, emotional, and academic development, with negative consequences for the families of the children, their community, and their society [18]. There is a wealth of research demonstrating that children with hearing loss who are identified earlier and receive early intervention have better outcomes than those with later detection and treatment [26,27,28,29,30]. Recent large-scale, population based, longitudinal studies provide convincing evidence for the positive long-term effects of universal NIHS programs on language, cognitive, and academic development of children and adolescents. For example, the Australian Longitudinal Outcomes of Children with Hearing Impairment (LOCHI) study [31,32] demonstrated that the earlier treatment with hearing aids or cochlear implants was started, the better the speech, language, and functional performance outcomes of children who are deaf or hard of hearing. Similarly, a British study showed better reading comprehension of a cohort of teenagers who received universal newborn hearing screening compared with a cohort of similar children who had not [12].

Both of the WHO resolutions about hearing loss have addressed this issue by urging WHO member states to develop, implement, and monitor screening programs for early identification of ear diseases and hearing loss in infants and young children [16,22]. Universal newborn and infant hearing screening (NIHS) programs have also been called for in many national and international position statements and guidelines, including, but not limited to: the National Institutes of Health [33], the American Academy of Pediatrics [34], the Joint Committee on Infant Hearing [35,36,37], and the European Consensus Development Conference of Neonatal Hearing Screening [38]. Essential quality criteria for NIHS programs according to these guidelines are the following:

1. A universal newborn hearing screening program should involve at least 95% of the newborns in a region.
2. Newborn hearing screening procedures should target the detection of at least all infants with bilateral hearing loss of ≥35 dB in the better hearing ear.
3. The proportion of babies who fail the newborn hearing screen and are referred for formal audiological testing should not exceed 4%.
4. The false negative rate for babies who passed the screening despite a substantial hearing loss should approach zero.
5. At least 95% of the babies failing the screening should be followed up with audiological diagnostics and, in cases of confirmed hearing loss, receive appropriate treatment.
6. The follow-up and access to diagnosis and treatment should be well organized, so that parents whose babies have failed the hearing screening can be referred directly to medical and audiological service centers that are qualified to provide accurate diagnostics and appropriate treatment for infants.
7. The audiometric diagnostics should be completed before 3 months of age and treatment for those with hearing loss should be initiated before 6 months of age.
8. NIHS programs should include a data collection system to ensure that the program meets the criteria specified above and is capable of tracking babies who have failed or missed the screening.
9. Procedures and tools for training and supervision of the people carrying out hearing screening should be implemented to ensure that screening is properly done.

Overarching principles of NIHS programs have also been published by the WHO in 2010 [13]. Automated (i.e., automatically analyzed) otoacoustic emissions (OAE) and automated auditory brainstem response (AABR) are the most frequently used screening procedures. Because they differ in sensitivity, specificity, and cost, thoughtful consideration needs to be given to deciding which protocols are chosen, and whether and how to combine these methods in newborn hearing screening programs. These objective (“physiological”) methods are valid and feasible even for low- and middle-income countries, as demonstrated by pilot projects and NIHS programs supported by non-governmental organizations [39].

Affordable, portable, easy-to-use hearing screening technology is available. There are also emerging technologies that make it possible to use wireless bi-directional data transfer for both screening and diagnostics after a failed hearing screen, with telehealth supervision from an expert screening centre [40,41,42]. Screenings are most often performed in maternity clinics, but may be done in other settings; for example, as community-based infant hearing screening programs in screening camps [43] or at primary health care immunization clinics [44,45], or integrated into public health care programs, with home screenings or parents visiting well baby clinics [46]. Parents of babies who have failed the screening need to be referred to audiological service centres where formal audiological diagnostics can be done and children who are diagnosed with hearing loss can be referred to appropriate treatment services (often referred to as early intervention).

The prevalence of neonatal permanent hearing loss is reported globally to be about 0.5 to 5.0 per 1000 infants, and in some low- and middle-income countries even higher [13]. Nearly two thirds of neonatal hearing loss affects both ears, while one third occurs unilaterally. The prevalence figures for hearing loss in newborns depend on factors such as the quality of the data assessment and the extent to which newborn hearing screening programs are established in a country or region. Prevalence figures tend to increase if the screening covers unilateral or mild hearing loss and where consanguine parenthood is frequent [47].

For neonates who have one or more risk factors for neonatal hearing loss, the prevalence is much higher—from 1% to 3% [48]. Risk factors for neonatal and childhood hearing loss have been repeatedly discussed by the US-based Joint Committee on Infant Hearing [36,37]. Based on this information and other sources, risk factors include:

- Genetic factors account for at least 40% to 60% of permanent childhood hearing loss and occur more frequently in cases of parental consanguinity [18,47,49,50]
- Family history of permanent childhood sensorineural hearing loss
- In-utero infection of mothers (e.g., cytomegalovirus, rubella, herpes, syphilis, and toxoplasmosis)
- Syndromes which may be associated with hearing loss (Down, Waardenburg, Pendred, Jervell, Lange–Nielson), or stigmata and findings associated with such syndromes (such as white forelock for Waardenburg syndrome)
- Craniofacial malformations, including those that involve the pinna and/or ear canal, such as ear tags, ear pits, and temporal bone anomalies
- Diseases or conditions that require extended neonatal intensive care
- Persistent pulmonary hypertension associated with mechanical ventilation, and conditions requiring the use of extracorporeal membrane oxygenation
- Elevated levels of hyperbilirubinemia
- Exposure to ototoxic medications (aminoglycosides, loop diuretics)
- Postnatal infections associated with hearing loss such as bacterial meningitis

## 4. Newborn and Infant Hearing Screening as Part of a National Plan on Ear and Hearing Care

To improve the situation of people with permanent hearing loss, countries should have a strategic plan that takes into consideration the demographic profile, requirements, and resources of the country. A manual on planning and monitoring of national strategies for ear and hearing care as published by the WHO [21] outlines strategies for the successful implementation of NIHS programs. It recommends advocacy as the first step, in other words, activities to raise awareness among politicians, health care professionals, funding providers, and the general public to get a political commitment and to mobilize the resources required. The WHO further recommends that member states establish national programs of ear and hearing health under the guidance of their ministries of health. WHO also recommends that a national ear and hearing health coordinator should chair a national committee for ear and hearing health. The committee should include all groups of professionals who deal with ear and hearing care as well as other key stakeholders.

The national committee should address, and possibly create task forces to deal with issues such as technology, training, infrastructure, equipment, finance, and advocacy. It should also guide, organize, supervise, and monitor the NIHS program. As part of this effort, relevant tools, standard operating procedures, and information materials should be developed. The standard operating procedures should include how, where, and when babies with a positive screening test should be referred [21]. Procedural criteria need to be specified about how many repetitions of a test still count as primary screening, and whether and how often babies should be re-screened. Periodic (quarterly, semi-annual, or annual) process indicators such as the number and distribution of neonates and infants screened for ear diseases and hearing loss (total and per 100,000 population) should be defined to monitor and evaluate the NIHS program [21]. 

If a comprehensive newborn hearing screening program is not yet operational, a situation analysis of needs and resources, as well as a strengths, weaknesses, opportunities, and threats (SWOT) analysis, should be performed, optimally by using the Ear and Hearing Care Situation Analysis Tool of the WHO [20]. This tool includes items that assess the situation of the NIHS, for example: Is there a government-led infant hearing screening program? Who leads the program? Which parts of the country or areas are covered by it? What percentage of the population is covered by the program? If there is no program, is there any government-led neonatal or infant screening program for congenital diseases? Is the NIHS program a stand-alone program or part of a broader activity? The tool also asks for a more general analysis that includes, for example, the allocation of a budget for ear and hearing care in the ministry of health, the provision of ear and hearing care services by the government, the private sector and nongovernmental organizations that provide hearing health care, the embedding of ear and hearing care in the public health sector, and whether there is a system of personal child health records [20].

## 5. Further Actions Regarding NIHS Programs

If not already done, all countries should establish national committees for ear and hearing health, including a newborn hearing screening program. A part of this program should include the prevention of neonatal hearing loss as mentioned above and described in the WHO publication on childhood hearing loss [18]. 

For situations where primary prevention of permanent infant hearing loss is not possible, NIHS programs can be an effective secondary prevention strategy that ensures detection and treatment of childhood hearing loss as early as possible. Early intervention using a family-centered, multidisciplinary approach that includes audiological, medical, therapeutic, rehabilitative, and pedagogical components and focuses on the importance of the family for child health can often prevent hearing loss from becoming disabling in its nature.

The 2017 WHO resolution calls for the preparation of a World Report on Ear and Hearing based on the best available scientific evidence. The resolution also urges member states “…to collect high-quality population-based data on ear diseases and hearing loss in order to develop evidence-based strategies and policies” [22]. Hence, ministries of health and national committees should regularly collect data about the current situation of ear and hearing care in the country and the status of hearing screening programs. Data collection may be difficult in countries with regional instead of national newborn hearing screening programs because the coverage and quality of the NIHS programs may differ between the various regions, states, or provinces. Such problems may be more severe in countries where only limited newborn hearing screening is being done–predominantly in hospital-based pilot projects, or in time-limited NIHS studies as have been done in several African countries such as Nigeria and Algeria [39].

## 6. Suggestions for Moving Forward

Disabling hearing loss is a global problem that is far from being solved, despite increased awareness among professionals and expert groups. This “invisible disease” is still underrepresented in the public and political awareness. As rising prevalence and growing burden related to hearing loss (as measured by YLDs), together with economic and sociodemographic indicators for different countries and regions suggest, a focus on prevention and treatment of childhood hearing loss would be very effective in reducing the burden of hearing loss in countries with lower levels of economic prosperity and sociodemographic indices [7].

Wilson and his colleagues [7] proposed a comprehensive worldwide initiative for disabling hearing loss similar to VISION 2020, which was a campaign that effectively reduced the burden of disabling vision loss. A similar global initiative aimed at reducing the prevalence and burden of disabling hearing loss may involve a global alliance where stakeholders with multiple backgrounds could cooperate and advocate for actions towards the common aim of raising awareness and promoting public health activities that would ameliorate the negative impacts of childhood hearing loss. A global alliance for hearing has been proposed by members of the WHO prevention of deafness and hearing loss program [8]. Examples of the effectiveness of WHO-coordinated alliances under the coordination of the WHO exist, such as the Violence Prevention Alliance [51]. One of the aims of such an alliance for hearing health should be the promotion of high-quality NIHS programs.

As an important step towards such an alliance, WHO has recently established the World Hearing Forum, a global advocacy network of stakeholders including representatives of governments, nongovernmental organizations, disabled people’s organizations, development agencies, academic institutions, and professional societies promoting ear and hearing care worldwide and aiming to foster the implementation of resolution WHA 70.13 (http://www.who.int/deafness/wolrd-hearing-forum/en/). This forum may also be helpful in the implementation of national early hearing detection and intervention programs by supporting advocacy and resource allocation.

The effectiveness of NIHS programs is expected to be increased by governmental mandates, guidance by the ministry of health, presence of national committees with broad stakeholder involvement, central organization of the screening, clear definition of benchmarks and target parameters, creation of tracking systems with bi-directional data transfer, easy accessibility of pediatric audiological services and rehabilitation programs, and opportunities for discussing complex cases with boards of experts. In low-income countries, community-based universal screening of infants during routine immunization clinics may be the most effective and high-throughput NIHS setting [44,45]. Not only has this setting been shown to yield the best outcome, but is also the most cost-effective option (US$ 7.62 in a study in Lagos, Nigeria [45]. Spreading internet connectivity and the digitalization of medicine will contribute to increased use of telehealth for audiology assessments, fitting of hearing devices, and providing early intervention. Training of health professionals, audiologists, and community health workers in the provision of screening and audiological services, as is done currently by, for example, the London School of Hygiene and Tropical Medicine, is further needed in middle- and low-income countries. 

There is a great potential for increasing the accessibility of treatment by reducing its costs, for example by changes in service provision, bulk purchasing, and new and economical hearing aid and cochlear implant technologies. Recent cost-effectiveness analyses have revealed that both hearing screening programs and subsequent treatment with hearing aids or other means were cost-effective in a range of countries (summarized in [7]).

## 7. Conclusions

The growing prevalence of disabling hearing loss and the associated increase in the global burden of hearing loss call for action that includes the implementation and improvement of NIHS programs. Such programs should be part of national programs for ear and hearing care. Regular and standardized data collection is necessary to better understand what is being accomplished and how to improve program effectiveness. Newborn hearing screening and follow-up treatment has been shown to be an excellent investment of resources. Nevertheless, adequate funding and resource allocation for these programs is needed, in particular for screening centers which provide tracking of babies and program monitoring. The foundation of a global alliance on hearing as proposed by the WHO could support the implementation, standardization, and supervision of national screening programs. We agree with the concluding sentences of Wilson et al. [7]: “The burden of hearing loss is higher than ever and is growing largely unabated. However, the capacity to prevent and treat hearing loss is growing as well. Economies are improving…; costs for prevention can be stunningly low; an unprecedented potential exists for reducing the costs for treatments... Now is a highly propitious time to tackle the burden with full force. The opportunities have never been greater, and the need has never been greater.”
